# “*Time lost is clot resolution lost*”: the neglected perspective of the therapeutic time window for ischemic stroke

**DOI:** 10.3389/fneur.2023.1177609

**Published:** 2023-05-24

**Authors:** Manuela De Michele, Svetlana Lorenzano, Lucia Bertuccini, Francesca Iosi, Danilo Toni

**Affiliations:** ^1^Emergency Department, Sapienza University of Rome, Rome, Lazio, Italy; ^2^Department of Human Neurosciences, Sapienza University of Rome, Rome, Lazio, Italy; ^3^Core Facilities, National Institute of Health (ISS), Rome, Lazio, Italy

**Keywords:** stroke, thrombus, therapeutic window for stroke, clot, intravenous thrombolysis (IVT)

“Time is brain,” a mantra adapted from the cardiological “time is muscle,” was first proposed 30 years ago (1), and it is still a valid concept, stressing the need for urgent intervention in acute ischemic stroke (AIS). The suffering but still viable neurons residing in the ischemic penumbra are at high risk of being included in the necrotic core over time. Hence, “time is brain” was also translated into the statement “time lost is penumbra lost,” highlighting even better the concept of the penumbral tissue lost during the therapeutic time window. In fact, it is well established that the efficacy of both intravenous thrombolysis (IVT) and mechanical thrombectomy (MT) gradually declines and the chance of recanalization and of reaching a good outcome is much higher during the 1st h after stroke onset, the so-called “golden hour” (2). More recently, the wider use of advanced neuroimaging made it clear that this evolution takes place at different paces, depending on the collateral circulation status (3).

Another issue to that rates of near-complete or complete recanalization of LVO in the AIS amount to a maximum of 32% with IVT (10–15 and 25–50% for internal carotid artery and proximal middle cerebral artery occlusion, respectively) (4, 5) and of 56–59.9% with MT (6). Therefore, for patients with LVO, bridging therapy is recommended (7).

Reasons for this recanalization “resistance” are not completely known. Clot burden, good collaterals (that can deliver more rTPA in the clot via backflow), timing from stroke onset, and thrombus composition have been advocated (8). In case of recanalization failure after MT, other important determinants are the pressure gradient across the thrombus and the stickiness of the thrombus itself (due to the combined force of friction and adhesion on the vessel wall) (9). Notably, it has been observed that the achievement of recanalization with a single thrombectomy device pass, the “first-pass effect,” is associated with a better outcome (6).

In 2014, in a single-center prospective study, Muchada et al. (10) showed that the effect of IVT on early recanalization detected by transcranial doppler sonography declined over time. Treatment initiation after 270 min was an independent predictor of lack of recanalization in distal MCA occlusion, whereas there was a trend toward lower recanalization in proximal MCA occlusion treated after 90 min. In the related editorial comment, Tsivgoulis and Alexandros (11) proposed the motto “time is clot” for thrombolytic therapy. After 1 year, Kim et al. (12) reported a linear inverse relationship between time from symptom onset to treatment and the degree of thrombus resolution after rtPA administration, assessed by a thin section non-contrast computed tomography scan performed at 1 h after IVT. In the same study, it was observed, by using an animal model of stroke, that the effect of rTPA depended on the thrombus age (12). The authors concluded their study by reviving the sentence “time is clot” for thrombolytic treatment but it did not gain a foothold and was early neglected. Afterward, growing evidence from research on retrieved thrombi during MT has been brought back into the spotlight, with the occluding clot as one of the main actors of the acute phase of IS.

It is now clear that, whether it has an atherothrombotic or an embolic origin, the clot is a tissue with specific characteristics evolving over time, whose probability to be resolved by thrombolytic therapy or removed by MT quickly declines pari passu (12). In this perspective, two variables are particularly relevant: length of thrombus and its composition.

Thrombi exceeding the length of 8 mm seem to have almost no chance to be recanalized by IVT (13).

Recent data on the composition of retrieved thrombi suggest that stroke clots can be categorized into platelet-rich thrombi and red blood cells (RBC)-rich thrombi with, in the middle, a wide range of more heterogeneous thrombi having a mixed coexisting platelet-rich and RBC-rich areas (14, 15).

RBC-rich clots and areas consist of densely packed RBC surrounded by a thin fibrin network, while platelet-rich clots and areas are much more complex and organized with dense fibrin strings, von Willebrand factor (VWF), platelets, DNA from neutrophil extracellular traps (NETs), and sparse leukocytes. Interestingly, networks of extracellular DNA and leukocytes have been more frequently found within the platelet-rich areas and at the interface between the platelet-rich and the RBC-rich areas but not within the RBC-rich areas (15).

Although it is not possible to analyze the histological characteristics of clots lysed and dissolved by rTPA, RBC-rich thrombi appear more susceptible to be lysed and more easily to be retrieved by thrombectomy devices compared to the other types of thrombi (16, 17). RBC-rich clots are also associated with non-cardioembolic stroke and with hyperdense MCA sign on non-contrast CT scan or blooming artifact on brain MRI (14, 18). In 2021, Gunning et al. demonstrated that fibrin-rich thrombi had a significantly higher coefficient of friction between the clot and the vessel wall than RBC-rich thrombi, thus contributing to the difficulty in retrieval by MT (19). In this regard, it has been observed that clots retrieved in earlier passes have higher RBC content in comparison with clots retrieved in later passes that are conversely, rich of fibrin, platelets, and other components (20). Interestingly, in this study, the extracted clot area was larger in the earlier than in later passes (20), supporting the fact that RBC-rich thrombi and RBC-rich areas inside the same thrombus are less sticky and more easily retrievable compared to fibrin-rich thrombi and fibrin-rich areas. However, it is not possible to rule out a direct effect of passes on the clot itself.

By using scanning electron microscopy (SEM), it is possible to identify two different time-related structural thrombotic patterns, one formed by dense fibrin mesh with sparse cellular elements suggesting a matured clot and another one characterized by looser fibrin strands and intact RBC suggesting a fresh and evolving clot (21). Figure 1 clearly shows these two patterns from the same thrombus retrieved by the proximal MCA of an adult patient treated in our center with bridging therapy 3 h after symptoms onset and analyzed by SEM.

**Figure 1:**
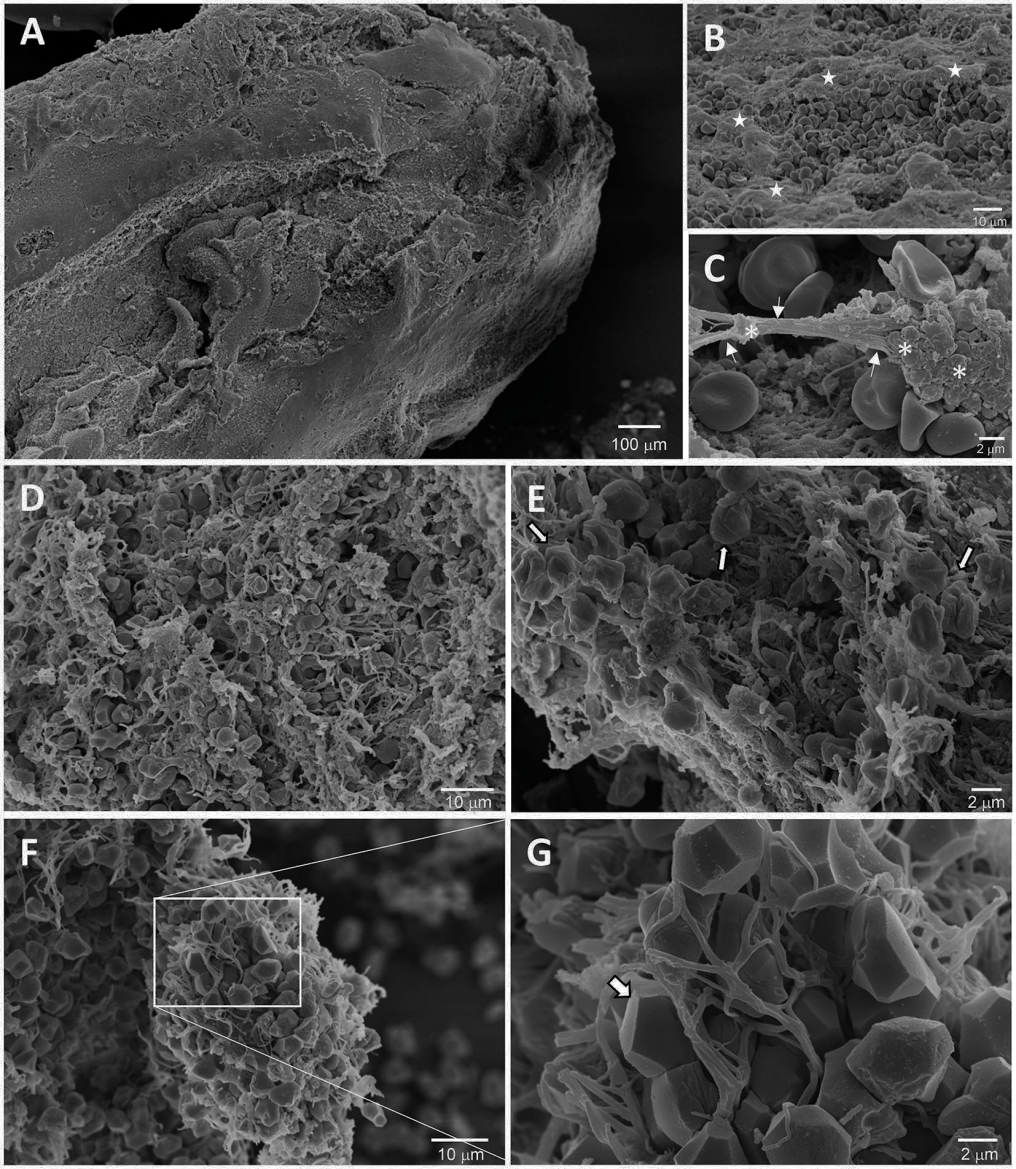
SEM micrographs of an arterial thrombus retrieved from a stroke patient. **(A, B)** Clot shell was characterized by a very thick meshwork of fibrin and platelet aggregates (**B**, white stars) that envelope erythrocytes with the typical biconcave shape. **(C)** High magnification of bundles of fibrin fibers (arrows) that protrude from strongly amassed platelets (*). **(D–G)** Inside portion of the thrombus displaying phases of the progressive clot contraction. **(D)** An area rich in partially compressed erythrocytes and platelet aggregates from which fibrin bundles protruded. **(E)** A partially compressed zone in which are clearly visible different intermediate shapes of erythrocytes scratched by the fibrin fibers compression (white arrows) (initial contraction of the clot). **(F)** A more compressed area of the clot in a more advanced phase of compression in which the erythrocytes have the typical polyedrocyte shape. **(G)** High magnification of the squared areas in **F** (arrow: polyedrocyte). SEM, Scanning Electron Microscopy.

Interestingly, SEM and immunohistological analysis of retrieved large vessel occluding thrombi have shown a common outer shell formed by densely compacted fibrin network, VWF, and aggregated platelets resistant to rTPA-mediated thrombolysis as compared to the inner core mainly formed by looser fibrin mesh and RBC (22). The fibrin shell thickness seems not to be dependent on patients' characteristics, pre-thrombectomy treatment, and stroke pathophysiology; the outer shell is more resistant to rTPA not only for its compaction and particular structure but also because platelet-derived direct inhibitors of tPA accumulate in it. Platelets play a fundamental role in the outer shell formation (22).

Although most of the available data on the evolution of thrombus composition come from venous and pulmonary embolism and *in vitro* studies, it is well recognized that arterial thrombus composition changes over time (14, 23).

Mechanisms of thrombosis are the result of an extremely complex interplay between endothelial cells, platelets, leukocytes- and platelet-derived microparticles, VWF, and coagulation factors (24, 25). High shear rate of blood is also an important contributor to arterial thrombus formation and propagation (24). Being such a highly dynamic process, clot formation in humans is difficult to study *in vivo*. However, studies on thrombus formation in live animals by using intravital microscopy and genetically altered mice (i.e., VWF null mouse) (25) confirmed data from *in vitro* experiments and shed light on the role of platelets and fibrin that strictly cooperate with the developing thrombus in the first 60 min of vessel occlusion (25, 26).

In the initial phases of thrombosis, platelets become activated due to contact with an altered endothelium surface, tissue factor, binding Factor VIIa, and active Factor IX and X that bind to Factor II to form thrombin. Thrombin transforms fibrinogen in fibrin and polymerizes fibrin monomers in fibers that entrap activated platelets and RBC (24). At this stage, the fresh thrombus presents a porous fibrin scaffold that rTPA can easily dissolve, especially because thrombin has not yet activated Factor XIII which catalyzes the formation of covalent bonds between adjacent fibrin subunits and the cross-links of alpha2-antiplasmin (an enzyme that participates to endogenous fibrinolysis) to fibrin, resulting in new fibers more resistant to lysis (27). Interestingly, it has been demonstrated *in vitro* that the scaffold and the fibrin network take less than 5 min in average to form (28), but the lateral aggregation of the protofibrils forming fibrin fibers reaches a plateau after 50 min (29). Clot contraction due to platelet–platelet, platelet–fibrin(ogen), and RBC–fibrinogen interaction stabilizes the clot by forcing platelets and fibrin on the external surface of the thrombus and by inducing the deformation of RBC into densely packed polyhedrocytes (30). At the same time, the crosstalk between activated platelets and leukocytes promotes further stability of the thrombus through the release of NETs by activated neutrophils (31). NETs promote fibrin deposition, bind VWF, favor platelet adhesion, and contain some factors (i.e., tissue factor) that make them procoagulants. As a result, over time, the thrombus quickly becomes a dense and compact structure adherent to the endothelial wall and is difficult to be lysed by rTPA and to be retrieved by thrombectomy devices (14).

The development of adjuvant treatment strategies targeting other clot components are actively under investigation and will likely improve the chance to lyse large vessel occluding clots (8).

## Conclusion

Stroke physicians and neurointerventionists should keep in mind that a clot is a vital and rapidly changing tissue. The sooner IV rt-PA is administered and endovascular thrombectomy is performed after stroke onset, the higher is the chance to dissolve the large vessel occluding thrombus and the better will be the outcome. That is a non-“neuro-centric” but “clot-centric” view of the ischemic stroke therapeutic time window. Indeed, time is not only brain but it is also clot: *time lost is clot resolution lost*.

## Author contributions

MD conceived the idea for the editorial and drafted the article. SL and DT critically revised the article. LB and FI analyzed the thrombus by using scanning electron microscopy and conceived the Figure 1. All authors contributed to the article and approved the submitted version.
